# Social isolation, rather than kinship, influences association behavior in toad tadpoles (*Bufo formosus*)

**DOI:** 10.1007/s10071-026-02090-0

**Published:** 2026-08-06

**Authors:** Kazuko Hase

**Affiliations:** 1 Research Center for Integrative Evolutionary Science, The Graduate University for Advanced Studies [SOKENDAI], Shonan Village, Hayama, 240‑0193 Kanagawa Japan; 2 Graduate School of Global Environmental Studies, Kyoto University, Yoshidahonmachi, Sakyo-ku, Kyoto, 606-8501 Japan

**Keywords:** Association preference, Aquatic environment, *Bufo formosus*, Kin recognition, Social experience, Toad tadpole

## Abstract

Experiences of social isolation alter the developing social behavior of animals. Association preference is an important behavioral tendency for amphibian larvae (tadpoles) that exhibit social aggregation as a means of predator avoidance. For tadpoles that exhibit social aggregation, the learning period during exposure to conspecifics could be crucial. This study investigated how water quality and social experience influence association preferences to unfamiliar conspecifics in *Bufo formosus* (Eastern-Japanese common toad) tadpoles. Using a 2 × 2 factorial design, subject tadpoles were reared either in tap or pond water and under group or solitary conditions. Binary-choice tests were then conducted to examine whether the tadpoles preferred siblings, non-siblings, or neither (neutral zone). No significant kin-biased association was detected in any treatment. However, social experience significantly affected the proportion of time spent in neutral zone. Solitary-raised tadpoles spent the most time in the neutral zone, whereas group-raised tadpoles spent more time on the effective-stimulus sides irrespective of water quality. It suggests that social isolation in early developmental stage reduces responsiveness to conspecifics. These findings indicate that social aggregation in *B. formosus* is shaped by social experience rather than kinship and aquatic environment.

**Supplementary Information:**

The online version contains supplementary material available at 10.1007/s10071-026-02090-0.

## Introduction

Social animals often show association preferences. During group formation, recognizing suitable partners is essential (Krause and Ruxton 2002). Because individuals rely on cooperation, they must discriminate among conspecifics (Henkel and Setchell 2018; Green et al. 2024). Since interactions with conspecifics are important for the formation of social relationships (Brandão et al. 2015; Medendorp et al. 2018; Groneberg et al. 2020), social isolation during early life is likely to influence association preference across taxa.

In many animal species, larvae benefit from grouping with conspecifics through predator avoidance (e.g., fish schools, Eggers 1978; Godin and Morgan 1985), thermal control (e.g., sea lion, Liwanag et al. 2014), and enhanced foraging efficiency (e.g., in birds, Camphuysen et al. 2004; in spiders, Rypstra 1989). Amphibian larvae (tadpoles) also form schools similar to those of fish and often exhibit kin-biased association (Waldman and Adler 1979; Waldman 1987). Because individuals hatch together within the same egg mass, they naturally learn the odors of their relatives through early association. Thus, tadpoles that possess kin recognition ability discriminate kin from non-kin by olfactory cues (Waldman 1985). From the perspective of inclusive fitness (Hamilton 1964a, b), competition among relatives should be reduced. In *Rana ornativentris*, to reduce kin competition, large-sized tadpoles avoid smaller siblings but approach smaller non-siblings for feeding (Hase and Kutsukake 2019). Furthermore, some forms of association preference are plastically determined rather than being entirely innate. In *R. ornativentris*, tadpoles raised with non-siblings no longer distinguish large-sized non-siblings from small-sized ones, indicating that rearing environment shapes recognition of preferred conspecifics (Hase and Kutsukake 2022).

To form social aggregations, interactions with conspecifics during the early developmental stage is likely an essential factor, regardless of whether kin discrimination occurs. It has been reported that the highly gregarious *Bufo bufo* tadpoles become more active in the presence of conspecifics (Griffiths and Foster 1998). Leu et al. (2013) indicated that *Litoria aurea* tadpoles can show direct attraction to conspecifics, but the strength of this tendency may depend on developmental and ecological context. In social animals, isolation from conspecifics during development can alter behavioral characteristics through changes in cognitive and neural processes. For example, developmental social isolation can have lasting effects on social behavior and neurochemical responses in zebrafish (Shams et al. 2018). Similarly, social isolation is associated with anxiety-like and depressive-like behaviors and reduced social interaction in mice (Shams et al. 2018). Therefore, social isolation may also affect social aggregation in toad tadpoles.

The Eastern-Japanese common toad (*Bufo formosus*) occurs in northeastern Japan and exhibits explosive breeding in early spring following hibernation (Hase and Shimada 2014; Matsui and Maeda 2018). Breeding typically occurs less than a week from mid-February to mid-March, and tadpoles complete metamorphosis within roughly six weeks (Matsui and Maeda 2018; Hase 2022). As in other Bufonidae species, the tadpoles are black, toxic, and form cohesive aggregations. Such grouping likely provides both predator dilution (Watt et al. 1997) and aposematic benefits through their dark coloration (Stynoski and Porras-Brenes 2022). Kin-biased association has been documented in *B. americanus* (Waldman 1982, 1986), *B. bufo* (Kiseleva 1989), *B. melanostictus* (Saidapur and Girish 2000; Eluvathingal et al. 2009) *Duttaphrynus scaber* (Gramapurohit et al. 2006); where individuals learn the odor of relatives from social interactions in the early developmental stage. If *B. formosus* shows similar kin-biased associations, learning is likely essential. However, no studies have examined association preferences in the absence of prior social experience—that is, after rearing in social isolation. Environmental factors, such as water quality, have also received little attention. Despite numerous studies, the mechanisms through which tadpoles develop social association preferences remain poorly understood.

In this study, I conducted behavioral experiments on *Bufo formosus* tadpoles to examine how the aquatic environment and social experience influence their association preferences toward unfamiliar conspecifics. If tadpoles recognize kin through olfactory cues, the microbe-rich environment of natural pond water may enhance the formation of odor cues compared with tap water. Microorganisms can contribute to the development of unique skin microbiome compositions in tadpoles, and such variation may be partly influenced by genetic factors. The microbiome may also contribute to individual-specific odor profiles, potentially affecting the acquisition of olfactory cues used in social recognition. To test this, larvae were reared in either tap water or pond water. To assess the effect of social experience, tadpoles were raised either in sibling groups or solitarily. If kin-biased association in *B. formosus* depends on early social experience, group-reared tadpoles are expected to show stronger preferences for sibling stimuli than solitary-reared tadpoles. Furthermore, social isolation may reduce responsiveness to conspecifics regardless of rearing water type. Using a 2 × 2 factorial design with two water types and two social conditions, I tested whether *B. formosus* tadpoles possess kin-biased association, and whether microbe-rich environments facilitate odor-based learning. The test arena was divided into three compartments: the effective sibling side, the effective non-sibling side, and a neutral zone. Time allocation in the three compartments was recorded using a real-time tracking program. First, I compared the time spent near the sibling and non-sibling stimuli to determine whether kin-biased association occurred. Second, I examined how social experience, water type, or their interaction influenced the tadpoles’ social tendencies by comparing time allocation in the three compartments.

## Materials and methods

### Animals

**Fig. 1:**
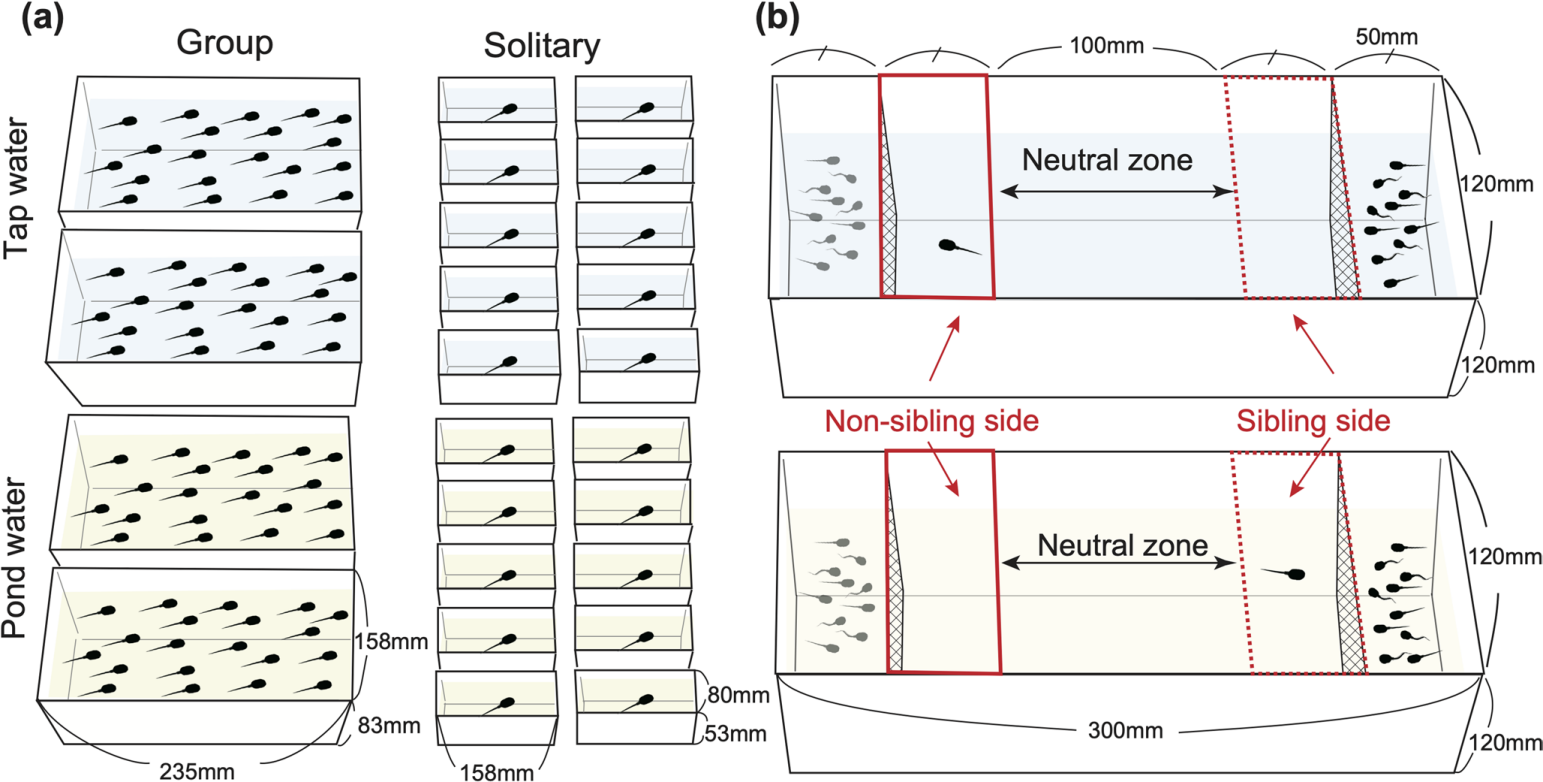
Diagram of the four rearing treatments (**a**) and the binary-choice test arena (**b**). Tap water was dechlorinated, and pond water was filtered through a 100-mesh polyethylene net. Water temperature was maintained at 18 ℃. Group- and solitary-reared containers were supplied with 1.5 L and 0.2 L of tap or pond water, respectively, which was changed every two days. For the binary-choice tests, the tank was filled with 1.5 L of water. Subjects could perceive visual and chemical cues from stimuli across polyethylene nets (strings: 0.1 mm diameter; mesh: 13–14 threads/cm²). The arena was divided into four equal parts, with the two ends designated as the stimulus sides and the center designated as the neutral zone. Dashed and solid line areas indicate quarters of the arena corresponding to the effective “Non-sibling stimuli side” and “Sibling stimuli side,” respectively

In April 2021, four egg strings (about 30 cm which contains 100–150 fertilized eggs) were collected from four separated puddles on the grounds of Botanical Gardens, Graduate School of Science, the University of Tokyo, Tochigi Prefecture, Japan. Larvae derived from each string were treated as a different sibling cohort in the rearing experiment. In the laboratory, after embryo hatching (developmental stage 22, Gosner 1960), the larvae were transferred to rearing containers before they started interacting with each other, i.e., swimming and feeding. The four rearing treatments were divided according to whether the water environment was tap water or pond water, and the social environment in which the larvae were reared, group (*N* = 20) or solitary (*N* = 1): tap water and group (hereinafter, Tg); tap water and solitary (Ts); pond water and group (Pg); pond water and solitary (Ps). Note that all groups are sibling groups derived from the same clutch. For use with subjects and stimulus, two Tg, two Pg, and six to ten Ts and Ps were prepared per clutch. Exact container sizes and social conditions, as well as the amount of environmental waters, are shown in Fig. 1a. Tap water was dechlorinated water. Pond water was transported from Ichijiro Pond at the campus of the University of Tokyo, and the water characteristics are the same as in the previous study (Hase 2022). Individuals were reared for two weeks in a large incubator at 18 °C, 12:12-h dark: light cycle with a sufficient supply of fish food pellets containing vegetable stock as the main component (PLECO; Kyorin, Hyōgo, Japan). And Individuals reaching the 30–35 developmental stage (Gosner 1960) were used in behavioral experiment.

## Behavioral experiment

To examine association preference of *B. formosus* tadpoles, the four types of choice tests were designed according to previous studies (Hase and Kutsukake 2022; Hase 2023). The differences in the four tests reflect the differences in the four rearing treatment types. A common scheme was used to verify kin recognition ability and the influence of the rearing conditioning in *B. formosus* tadpole. Diagram of the arenas where the binary-choice tests were conducted is shown in Fig. 1b.

The arena was divided into four equal sections, and 10 stimulus individuals, 10 siblings or 10 non-siblings were placed. The time spent by the subject tadpoles were measured on three compartments: effective-sibling side, neutral zone, and effective-non-sibling side (Fig. 1b). The subject individuals, placed quietly in the center of the arena from their rearing container using a perforated spoon, were captured by a camera (HCV720-M; Panasonic, Osaka, Japan). Each trial was conducted with 5 min of acclimation followed by 30 min, then the process was repeated with left and right turns to eliminate bias, for a total of 60 min. All subjects were tracked by UMAtracker (Yamanaka and Takeuchi 2018), and the time spent in each compartment was measured in 1-second intervals. During the test, water was used under conditions similar to those under which the subjects were raised. For example, when individuals raised in pond water were tested, the stimulus individuals were also grown in pond water, and the arena was filled with pond water (Fig. 1b). Four types of subjects were used in accordance with four different rearing conditions. All stimuli (siblings and non-siblings) were unfamiliar, i.e., stimuli tadpoles had been raised separately in a container from subjects. A total of 90 individuals were used for subject tadpoles, and each tadpole was tested only once. Raw data of the four types of choice test were shown in supplementary table S1.

### Data analysis

Statistical analyses were conducted using R version 4.3.1(R Core Team 2023). To access whether tadpoles exhibit kin-biased association, I compared the time spent on each effective stimulus side (out of 3600s), sibling and non-sibling, using a paired t-test. Differences were considered significant at *p* < 0.05.

Next, to examine the effects of rearing conditioning (social experience and water type) on the subject tadpole behavior, I analyzed the proportion of time that each individual spent in each zone of the test arena (sibling side, neutral zone, and non-sibling side). To avoid treating consecutive seconds as independent observations, analyses were conducted at the individual level (time spent in each zone divided by total observation time, e.g., if the time spent in a zone was 1268s, the score, which was translated to 1268s / 3600s = 0.352). Because the variance associated with clutch identity was estimated to be zero, clutch identity was not treated as a random effect. Instead, clutch was included as a fixed factor in the analysis. Linear models (LMs) with Gaussian error distributions were used to examine the effects of social experience (group or solitary rearing), water type (tap water or pond water), their interaction, and clutch identity. Separate models were fitted for the sibling side, neutral zone, and non-sibling side, using the proportion of time spent in each zone as the response variable.

## Results

**Fig. 2:**
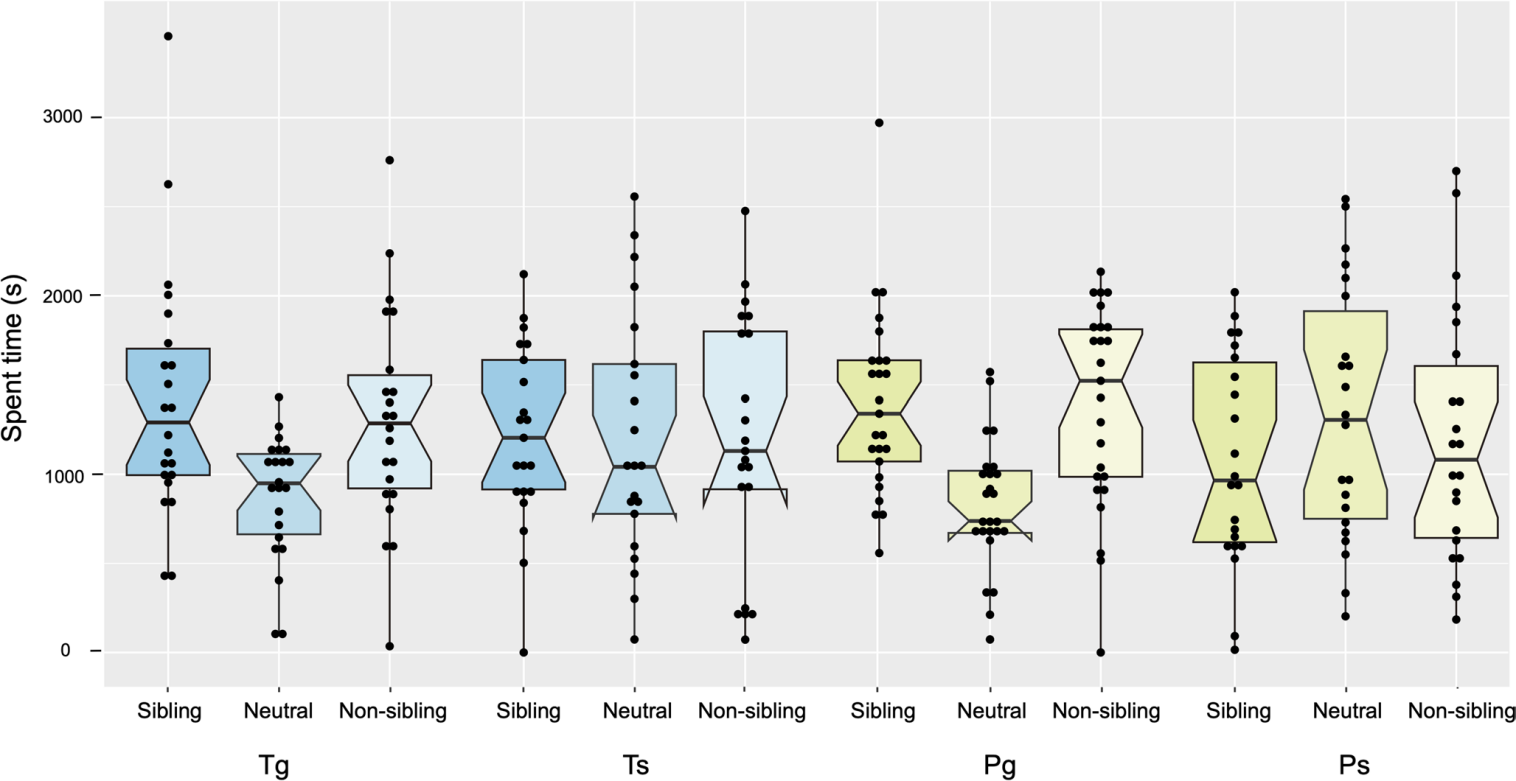
A notched box plot with overlaid raw data showing the time spent in three compartments: effective sibling side, neutral zone, and effective non-sibling side, across four types of rearing treatments. Rearing treatments for the subject tadpoles are tap water with group rearing (Tg), tap water with solitary rearing (Ts), pond water with group rearing (Pg), and pond water with solitary rearing (Ps), shown from left to right on the x-axis. Within each box, horizontal lines represent the median values. The boxes extend from the 1st to the 3rd quartile, the whiskers indicate the range up to 1.5 times the interquartile range beyond the boxes, and the notches represent approximate 95% confidence intervals for the median values

The results of the binary-choice tests are presented in Table 1; Fig. 2. Across all rearing treatments, no significant differences were observed between the time spent on the sibling and non-sibling effective sides. The results of the paired t-tests were as follows: Tg: t = 0.419, df = 21, *p* = 0.680; Ts: t = 0.129, df = 20, *p* = 0.899; Pg: t = 0.043, df = 24, *p* = 0.966; Ps: t = -0.505, df = 21, *p* = 0.619. These results provide no evidence of kin-biased association in the tested tadpoles.

**Table 1:** Summary of the binary-choice test between unfamiliar siblings and unfamiliar non-siblings. Rearing treatments for subject tadpoles. Water, type of water. Sociality, social treatments. Sample size, number of subject tadpoles. Spent time on the effective sibling side, the neutral zone, and the effective non-sibling side, (out of 3600s)

Rearing treatments			Sample size	Spent time on sibling side (mean ± SD)	Spent time on neutral zone (mean ± SD)	Spent time on non-sibling side (mean ± SD)
Water	Sociality	Abbreviation				
Tap	Group	Tg	22	1419.0 ± 702.2s	875.0 ± 352.3s	1306.0 ± 608.3s
Tap	Solitary	Ts	21	1213.3 ± 511.7s	1202.1 ± 696.9s	1184.6 ± 706.6s
Pond	Group	Pg	25	1393.3 ± 518.2s	822.2 ± 364.6s	1384.4 ± 568.3s
Pond	Solitary	Ps	22	1075.2 ± 576.9s	1332.0 ± 695.8s	1192.8 ± 692.2s

Linear model (LM) analyses revealed no significant effects of social experience, water type, their interaction, or clutch identity on the proportion of time spent on either the sibling side (social experience: t = -1.849, *p* = 0.068; water type: t value = 0.132, *p* = 0.895; interaction: t value = 0.460, *p* = 0.647; clutch identity: t value= -0.339, *p* = 0.7352) or the non-sibling side (social experience: t = -1.018, *p* = 0.311, water type: t value = -0.374, *p* = 0.709; interaction: t = 0.241, *p* = 0.810; clutch identity: t = 0.791, *p* = 0.431). In contrast, social experience significantly affected the proportion of time spent in the neutral zone (t = 3.155, *p* = 0.002), whereas water type (t = 0.298, *p* = 0.767), the interaction between social experience and water type (t = − 0.770, *p* = 0.444), and clutch identity (t = − 0.567, *p* = 0.572) were not significant. Tadpoles reared in isolation spent a greater proportion of time in the neutral zone than group-reared tadpoles, regardless of water type (Table 1).

## Discussion

The results of the present study indicated that social experience had a stronger influence on the association behavior of *Bufo formosus* tadpoles than either kinship or water type (Table 1; Fig. 2). Social isolation caused tadpoles to spend more time in the neutral zone and less time near the stimulus sides, whereas group-reared tadpoles remained closer to the stimulus sides. On the other hand, no significant differences were found in the time spent on the sibling versus non-sibling sides across all rearing treatments. Contrary to expectations, *B. formosus* tadpoles did not exhibit kin-biased association, unlike several related species reported previously (e.g., Waldman 1982, 1986; Kiseleva 1989; Saidapur and Girish 2000; Eluvathingal et al. 2009). Pond water and tap water differ in their chemical properties, and these differences could potentially alter the odor cues involved in kin-template formation. 

Nevertheless, water quality also did not affect association behavior regardless of social experience. In all, in the context of social aggregations in *B. formosus*, social experience appears to play a greater role than in exhibiting kin-biased association.

Since subject tadpoles from solitary containers spent more time in the neutral zone than those from group containers, this suggests that social isolation reduced responsiveness to conspecific stimuli regardless of kinship or water type (Fig. 2). This result suggests that social aggregation in *B. formosus* tadpoles depends on early social interactions with conspecifics during development, i.e., before the emergence of the hindlimbs (Gosner stage > 30, Gosner 1960). In tadpoles, social context can influence activity and aggregation tendencies, and conspecific presence has been shown to increase activity in aggregating species such as *Bufo bufo* (Griffiths and Foster 1998). The effect of social isolation observed in the present study is consistent with evidence from other taxa showing that early social experience can shape responses to conspecifics. Similar effects of early social experience have also been reported in fish: socially isolated larval zebrafish show enhanced social avoidance responses (Wee et al. 2022), and social isolation can alter neural circuits involved in processing conspecific cues and regulating avoidance behavior　(Shams et al. 2018; Groneberg et al. 2020). Social isolation may affect neural development and reduce social responsiveness in toad tadpoles, as shown in cichlid fish (Brandão et al. 2015) and in mice (Koike et al. 2009; Lander et al. 2017). However, because general locomotor activity was not measured independently in the present study, I cannot exclude the possibility that social isolation also affected overall activity levels. Future studies should include non-social control conditions to distinguish reduced social responsiveness from general changes in locomotion.

Regarding water environments, I predicted that tadpoles reared in pond water, which contains more microorganisms, would exhibit stronger odor-based differences between siblings and non-siblings than those raised in tap water. However, at least in the present study, this prediction was not supported. Indeed, no kin-biased association was observed under both pond and tap water conditions, regardless of social treatment. Why did *B. formosus* not exhibit kin recognition in the present study? The reasons for this can be discussed from two perspectives.

First, in the grouping behavior of toad tadpoles, the number of conspecifics may be more important than kin-biased association. The primary function of tadpole aggregation is likely predator avoidance. It has been proposed that the distinctive black coloration of these tadpoles serves as a warning signal to predators, which is enhanced by cohesive aggregation (Wells 2010; Stynoski and Porras-Brenes 2022). In this context, kin-biased association with siblings is not essential for effective aggregation. Some species may therefore achieve cohesive social aggregation without kin discrimination. Watt et al. (1997) showed that tadpoles are less likely to be predated upon when in larger groups. Additionally, a binary-choice test in Miyako toads (*B. gargarizans miyakonis*) indicated that group size is a critical factor (Hase 2023). In the present study, the same number of tadpoles (10 vs. 10) was used in the binary-choice tests, and no significant differences were observed (Table 1; Fig. 2). This suggests that, in black-colored toad tadpoles, group size may be more important than kinship in aggregation behavior.

Second, the absence of kin-biased association may reflect mechanistic constraints associated with the genetic background of the tadpoles (Green et al. 2015; Scott et al. 2022). Previous studies using *B. americanus* demonstrated the ability to discriminate unfamiliar siblings from familiar non-siblings (Waldman 1986). The experimental design of Waldman (1986) did not differ fundamentally from the present study, although it is possible that the siblings and non-siblings in previous studies had greater genetic divergences than those used in this study. In other words, it is highly likely that there were not enough differences in genetically determined odor traits to distinguish between siblings and non-siblings. This background pattern may be common across species in which kin recognition ability is present in some tadpoles but absent in others. For example, *B. boreas* did not exhibit kin-biased association (O’Hara and Blaustein 1982). The ‘odor of relatives’ in *B. formosus* and *B. boreas* tadpoles may not differ enough to allow sibling and non-sibling distinction. However, the absence of observed kin discrimination in *B. formosus* does not necessarily indicate a lack of kin recognition ability. They may instead use learned olfactory cues after metamorphosis, particularly during sexual maturation, for example, in the context of inbreeding avoidance (Waldman 2005).

Although no kin-biased association preference was observed in the present study, solitary-reared tadpoles spent more time in the neutral zone than group-reared tadpoles, suggesting that early social experience influences responses to unfamiliar conspecifics. Further studies are needed to clarify how social isolation affects social aggregation and whether these effects persist beyond the larval stage and influence behavior after metamorphosis in amphibians.

## Supplementary Information

Below is the link to the electronic supplementary material.

Supplementary Material 1

## Data Availability

All datasets generated during and/or analyzed during the current study are available in the supplementary material.
